# Identification of Potent Bioassay Guided Terpenoid and Glycoside Root Fractions of *Astragalus candolleanus* against Clinically Significant Bacterial Strains

**DOI:** 10.1155/2022/4584799

**Published:** 2022-04-28

**Authors:** Kandasamy Nagarajan, Roma Ghai, Garima Varshney, Parul Grover, Carlo Genovese, Floriana D'Angeli, Richa Goel, Thota Prasad, Muthusamy Kalaivani, Anil Kumar Teotia

**Affiliations:** ^1^KIET School of Pharmacy, KIET Group of Institutions, Delhi-NCR, Ghaziabad 201206, India; ^2^Faculty of Medicine and Surgery, “Kore” University of Enna, Contrada Santa Panasia, Enna 94100, Italy; ^3^Nacture S.r.l, Spin-off University of Catania, Via Santa Sofia 97, Catania 95123, Italy; ^4^Department of Human Sciences and Quality of Life Promotion, San Raffaele Roma Open University, Via di Val Cannuta 247, Rome 00166, Italy; ^5^Indian Pharmacopoeia Commission, Sector-23, Raj Nagar, Ghaziabad 201002, India

## Abstract

Antibiotic resistance represents one of the biggest challenges, and there is an urgent need for plant-based antimicrobial agents that enable managing this crisis effectively. In this work, we aimed to investigate the antibacterial activity of *Astragalus candolleanus* (*A. candolleanus*) hydromethanolic root extract against Gram-positive (*Bacillus subtilis* and *Staphylococcus aureus*) and Gram-negative (*Escherichia coli*, *Salmonella typhimurium*, *Pseudomonas aeruginosa*, *Klebsiella pneumoniae*, and *Kocuria rhizophila*) strains by the cup-plate method. The root was powdered and extracted with 70% methanol by cold maceration for 5 days. Preliminary phytochemical screening was performed with different solvents in the order of increasing polarity. Pure compounds were isolated by column chromatography and were characterized through liquid chromatography-mass spectrometry. Targeted predictions of the isolated compounds were also studied using Swiss Target prediction software and prediction of activity spectra for substances. The extract showed a broad zone of inhibition against pathogenic bacteria. Four pure compounds were isolated, of which a novel terpenoid compound has been identified as stemmadenine along with scillirosidin, cephalotaxine, and myxoxanthophyll. The structures of the isolated phytoconstituents were elucidated by spectral analysis. The four pure components isolated from the roots of *A. candolleanus* are suggested to be effective against tested pathogens. Overall results of drug design suggest that myxoxanthophyll is a promising bioactive compound endowed with antibacterial activity.

## 1. Introduction

Antibiotic resistance represents a serious problem for public health [1, 2]. Despite the enormous efforts to limit this phenomenon, an increasing number of antimicrobials, which were designed to kill or arrest the growth of bacteria, viruses, or fungi, are becoming ineffective, so antibiotic-resistance-related therapeutic failure is currently a real emergence worldwide [3–6]. This condition significantly affects our ability to prevent and treat infectious diseases promptly. In the last few decades, the discovery and development of novel anti-infective drugs have represented an active research area. Concerning this, natural compounds have historically been recognized as a rich source of anti-infective drugs, which provided penicillin in 1940, tetracyclines in 1948, and glycopeptides in 1955 [7]. This evidence promoted the study of natural products, considering a valid source of bioactive molecules that could help to manage this crisis effectively. *Astragalus rhizanthus* subsp. *Candolleanus* Benth. (synonym Rudravanti) (*A. candolleanus*) belong to the family Fabaceae, and it is a wild-growing herb widely spread in the Himalayas from Jammu & Kashmir to Uttarakhand provinces in India [8, 9]. *A. candolleanus* is endowed with several health benefits, such as immune-boosting, antiaging, and anti-inflammatory effects [10, 11]. This plant has also been useful in the treatment of blood and skin diseases, tuberculosis, and joint pains and as an antidiabetic medication [12–15]. A few of the most common bacteria that can cause complicated life-threatening infections like septicemia are *Escherichia coli* (*E. coli*), *Staphylococcus aureus* (*S. aureus*), *Bacillus subtilis* (*B. subtilis*), *Pseudomonas aeruginosa* (*P. aeruginosa*), *Klebsiella pneumoniae* (*K. pneumoniae*), and *Kocuria rhizophila* (*K. rhizophila*) [16]. Interestingly, a previous study showed the antibacterial and antibiofilm activities of *Astragalus angulosus* ethanolic extract against three Gram-positive strains (*Staphylococcus epidermidis*, *S. aureus*, and *Enterococcus faecalis*) and two Gram-negative strains (*E. coli* and *P. aeruginosa*) [17–19]. Albayrak and Kaya investigated the antibacterial and antifungal activities of four *Astragalus* species (*Astragalus gummifer*, *Astragalus microcephalus*, *Astragalus talasseus*, and *Astragalus acmophyllus*) endemic to Turkish flora. Specifically, they performed the disk diffusion assay against *P. aeruginosa* (low activity with respect to standard antibiotics tetracycline and oxacillin) and *Candida albicans* (no activity) [20]. Recently, Guo et al. demonstrated the antibacterial activity of *Astragalus membranaceus* ethyl acetate aerial parts extract. In particular, it was effective against the Gram-positive strain *B. subtilis* [21]. This activity could be related to the high concentration of flavonoids in the extract [22]. However, to date, only a few studies focused on the biological activity and chemical composition of *A. candolleanus* root extracts. Considering that literature data reported outstanding biological activity of the root extracts from other *Astragalus* species, we proposed to fill the knowledge gap on *A. candolleanus* by investigating the chemical profile and antimicrobial activity of its extract. After a thorough literature survey on *Astragalus* species with emphasis on its phytochemical screening in various parts of the plant (root, leaf, and fruit), the root extract was considerably found to be enriched with phytoconstituents [23–26]. Interestingly, it has been found that the root of *Astragalus mongholicus* is colonized by several bacterial species, which are able to modify the secondary metabolites of the plant. According to such a study, the biological properties and chemical features of a phytoextract can be the results of the interaction between bacteria and plants [27]. So, the present work aimed to investigate the antibacterial potential of *A. candolleanus* root extract against the most common bacteria involved in septicemia and, subsequently, identify the active phytoconstituents responsible for the biological activity. For this purpose, by column chromatography, the root extract was fractionated to isolate the various pure phytoconstituents. These pure components were elucidated using the mass spectrometry technique.

## 2. Materials and Methods

### 2.1. Chemicals

All the reagents were of analytical grade and purchased from Merck & Hi Chem Life Sciences except those mentioned elsewhere.

### 2.2. Plant Material

The roots of *A. candolleanus* were collected from Losar (32.4366°N, 77.7381°E) district of Himachal Pradesh. The plant's identity was confirmed by an acknowledged botanist, Dr. Sunita Garg, Emeritus Scientist, CSIR-NISCAIR Raw Material Herbarium and Museum, New Delhi (RHMD). A specimen voucher (NISCAIR/RHMD/Consult/2018/3253–54) was deposited at NISCAIR.

### 2.3. Preparation of Plant Extract

*A. candolleanus* roots were dried at room temperature and reduced to a coarse powder. About 20 g of the powdered root was extracted with 200 mL of methanol-water mixture (7 : 3 *v/v*) by cold maceration for 5 days. Afterwards, the mixture was decanted and filtered to get the crude extract. The extract was then concentrated under reduced pressure through a rotavapor (Buchi-R100), followed by drying on a desiccator.

### 2.4. Preliminary Phytochemical Screening

One milligram of the powdered roots was macerated individually in volumetric flasks containing different solvents, including dimethyl sulfoxide (DMSO), *n*-hexane, petroleum ether, chloroform, methylene chloride, acetone, ethyl acetate, methanol, ethanol, water, methanol : water (7 : 3 *v/v*), and *n*-butanol : acetic acid : water (BAW) (4 : 1 : 5 *v/v*). The powdered root extract along with different solvent systems was allowed to stand for 48 hours. After filtration, chemical tests allow the qualitative analysis of the extract [18, 28]. The presence of several chemical classes of compounds, such as alkaloids, glycosides, terpenoids, flavonoids, saponins, carbohydrates, lipids, volatile oils, steroids, phenols, tannins, gums, and mucilage, was determined. The chemical assays were conducted solely on the extracts without any hydrolysis.

### 2.5. Antibacterial Activity

The bacterial strains were purchased from the American Type Culture Collection Centre (ATCC) through an authorized vendor, Hi-Media Pvt. Ltd. Resources (reagents and apparatus) from the Indian Pharmacopoeia Commission, Ghaziabad, and used to carry out this research. The antibacterial activity of hydro-methanolic root extract of *A. candolleanus* was evaluated by the cup-plate method against *B. subtilis* (ATCC 6633), *E. coli* (ATCC 9637), *K. pneumoniae* (ATCC 10031), *K. rhizophila* (ATCC 9341) *P. aeruginosa* (ATCC 25619), *Salmonella typhimurium* (ATCC 1428), and *S. aureus* (ATCC 6538). Bacteria were cultured on nutrient agar media (Hi-media). The method of Ali *et al.* with some modifications was used for the antibacterial assay [19]. The 5 mm bores were made in the agar medium through sterile cork borers. 100 *μ*L of extract (100 mg/mL) in different dilutions (from 5 to 80 *μ*g/ml) was placed in the wells along with DMSO as a negative control. DMSO was diluted in a 1 : 100 ratio and did not affect bacterial growth. The activity of the natural extract was compared to that of the standard antibiotic gentamycin, in concentrations ranging from 5 to 40 *µ*g/ml. The agar plates were incubated for 24 h at 30–35°C. The parameters used for observation were the estimation of the inhibition zone of bacterial growth surrounding the wells. The unit for the diameter of the inhibition zone was taken in millimetres (mm).

### 2.6. Isolation of Constituents from *A. candolleanus* Roots

A column of 400 mm length with 500 mL reservoir capacity, 30 mm internal diameter, and 40 mm outer diameter was prepared with the wet packing method using silica (100–200 mesh size) as a stationary phase. The hydro-methanolic root extract was packed into the column and was eluted in a sequence from nonpolar solvents to polar solvents to obtain different pure fractions. The similarity profile of fractions was checked by thin layer chromatography, and similar fractions were identified. Pure components could be obtained in the case of root extract eluted with 70% *v/v* ethanol-water. The isolated and purified compounds were analyzed using Fourier-transform infrared spectroscopy (Shimadzu, IR Affinity-1) and liquid chromatography-tandem mass spectrometry (LC-MS-MS) (Agilent 6520).

### 2.7. Determination of Melting Point of the Isolated Compounds

The melting point is an intensive physical property that is characteristic of a specific compound. Thus, the melting points of the isolated compounds were determined to ensure their purity. All melting points were measured on a melting point apparatus (Accuma India Digital Melting/Boiling point apparatus).

### 2.8. Target Prediction of Isolated Compounds

The isolated compounds were subjected to Swiss Target Prediction (STP) (https://www.swisstargetprediction.ch/) [28] and Prediction of Activity Spectra for Substances (PASS) online bioactivity score software (https://www.way2drug.com/) [29–31] to understand the probable targets of these compounds.

## 3. Results and Discussion

After evaporation of the solvent, the screening for active phytoconstituents in the semisolid hydro-methanolic root extract of *A. candolleanus* showed the presence of alkaloids, glycosides, terpenes, lipids, volatile oil, gums, and mucilage.  Table 1 demonstrates the results of the phytochemical analysis: a pilot screening was performed using several solvents/solvent systems characterized by a different polarity.

**Figure 1:**
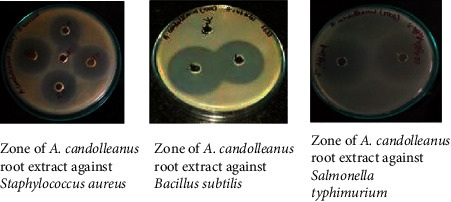
Representative image of the antibacterial effect of *A. candolleanus* extract against some tested bacterial strains.

**Figure 2:**
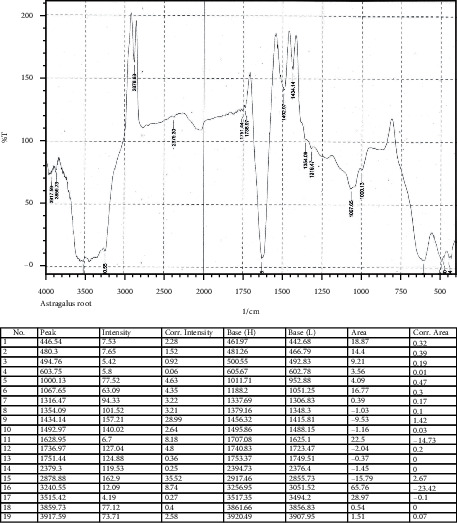
FTIR spectrum of *Astragalus candolleanus* root extract.

The hydro-methanolic root extract of *A. candolleanus* was able to inhibit the growth of all tested Gram-positive and Gram-negative strains. Specifically, as reported in Table 2 and Figure 1, the extract exerted the antibacterial activity already at the concentration of 5 *µ*g/mL; however, at the concentration of 10 *µ*g/mL, it produced a broader inhibition zone against *S. aureus* (28.6 mm), *S. typhimurium* (25.5 mm), *K. rhizophila* (24.6 mm), *K. pneumoniae* (21.8 mm), and *E. coli* (22.7 mm). Besides, at this concentration, *A. candolleanus* extract showed a strong growth inhibition for *B. subtilis* (41.2 mm) and *P. aeruginosa* (41.6 mm). These bacteria are seen to be mainly responsible for recurrent bacterial infections. The obtained results indicated that the hydro-methanolic root extract of *A. candolleanus* can be considered a promising antimicrobial agent, due to its broad zone of inhibition against pathogenic bacteria. Our results revealed that the extract was more efficient in counteracting the growth of Gram-positive strains compared to the Gram-negative ones, except for *P. aeruginosa*. In this regard, it is worth noting that Gram-negative bacteria are generally more resistant to the natural antimicrobial agents compared to the Gram-positive ones. This condition reflects the different composition of the cell wall between the two types of bacteria [32]. The bacterial cell wall is a multilayered structure that protects microorganisms from different environmental conditions and antimicrobial stress. Besides, it confers a characteristic shape and prevents cell rupture. In particular, the Gram-positive bacterial cell wall is formed by a thick layer of peptidoglycan, which is cross-linked with long anionic polymers called teichoic acids. Conversely, Gram-negative bacteria are endowed with a thinner layer of peptidoglycan, surrounded by an outer membrane containing lipopolysaccharides, extremely selective to the passage of xenobiotics [33, 34]. This structural organization constitutes an efficient barrier against external agents, making the Gram-negative related infections very difficult to treat [35]. Concerning the molecular aspect, there are numerous mechanisms of action through which antimicrobial agents act, including inhibition of synthesis of bacterial proteins, inhibition of cell wall synthesis, damage to the bacterial cell membrane, interference with DNA replication/repair, and their metabolic pathway [36]. The characterization of phytoconstituents from the roots of *A. candolleanus* allows the identification of pure compounds. The isolated and purified compounds were analyzed using FTIR and LC-MS-MS techniques. The results and the inference of the characterization of *A. candolleanus* root by FTIR are presented in Table 3. The melting points observed for isolated compounds are 168–170°C (169°C for reference) for scillirosidin, 151–155°C (153°C for reference) for cephalotaxine, 280–288°C (287°C) for stemmadenine, and 168–172°C (169°C for reference) for myxoxanthophyll [37–40]. The FTIR spectrum of *A. candolleanus* root extract is shown in Figure 2.

**Figure 3:**
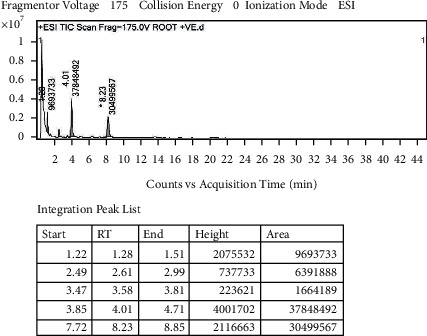
Mass spectral analysis of *Astragalus candolleanus* root extract by LC-MS-MS.

**Figure 4:**
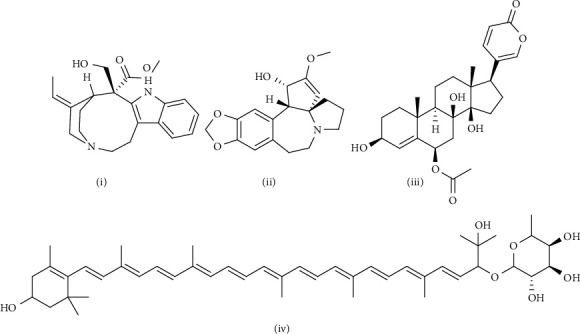
Structure of isolated compounds: (i) stemmadenine, (ii) cephalotoxin, (iii), scillirosidin, (iv) and myxoxanthophyll.

With the help of data and spectrum obtained from the LC-MS-MS technique, phytoconstituents present in the root extract of *A. candolleanus* have been identified. Four compounds have been isolated and identified using the data of peaks of mass spectral analysis (Figure 3): the terpenoid stemmadenine, the alkaloid cephalotaxine, the glycosides scillirosidin, and myxoxanthophyll (Figure 4).

**Figure 5:**
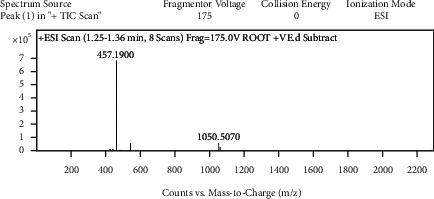
Mass spectra of peak 1.

**Figure 6:**
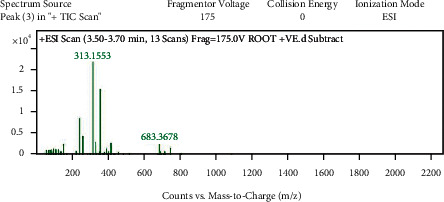
Mass spectra of peak 3.

**Figure 7:**
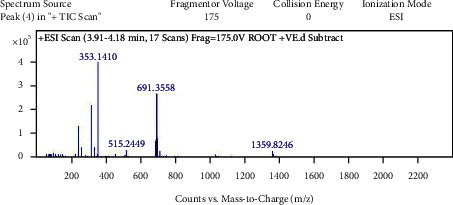
Mass spectra of peak 4.

**Figure 8:**
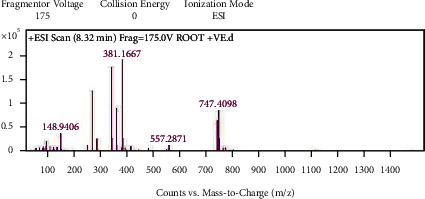
Mass spectra of peak 5.

Results of LC-MS-MS of *A. candolleanus* root extract are displayed in Tables 4–7. The LC-MS-MS spectra are given in Figure 3 and Figures 5–8. The compound identified from peak 1 is a glycoside—scillirosidin (molecular weight: 458.55 and empirical formula: C_26_H_34_O_7_). The compound identified from peak 3 is an alkaloid—cephalotaxine (molecular weight: 315.369 and empirical formula: C_18_H_21_NO_4_). The compound identified from peak 4 is a terpenoid—stemmadenine (molecular weight: 354.45 and empirical formula: C_21_H_26_N_2_O_3_). The compound identified from peak 5 is a myxol glycoside—myxoxanthophyll (molecular weight: 747.026 and empirical formula: C_46_H_66_O_8_).

According to earlier studies, terpenoids are found to be more effective against Gram-positive bacteria than Gram-negative bacteria due to their lipophilic properties. Monoterpenes preferentially affect the membrane structures by enhancing the permeability as well as changing the structural arrangement of proteins, producing interference inside the respiratory chain [41]. The natural or synthetic quinolone alkaloids have been found to block the action of topoisomerase enzymes, especially type-II variants, thus preventing nuclear replication [42]. Furthermore, it has been reported that some of the phenols can inhibit the enzymatic activity of the bacterial DNA gyrase by interacting with its ATP site [43]. Concerning the four pure components, stemmadenine, scillirosidin, cephalotaxine, and myxoxanthophyll, isolated from the roots of *A. candolleanus*, further studies should be conducted on these microorganisms to confirm their effectiveness as well as to elucidate the mechanism of action through which they exert the antibacterial activity. In response to bacterial infection, high levels of proinflammatory cytokines, such as interleukin-6 (IL-6), IL-8, IL-18, tumor necrosis factor-alpha (TNF-*α*), and anti-inflammatory cytokine (IL-10) were often found in infected patients. A decrease in IL-6 was associated with a better prognosis instead, and overproduction of IL-10 is considered the main predictor of severity and fatal outcome. In bacterial infections, proinflammatory and anti-inflammatory cytokines are a double-edged sword: on the one hand, they are essential for eradicating the pathogen, but their overproduction can cause tissue and organ damage [44, 45]. The isolated compounds were subjected to STP and PASS analysis to determine the probable biological activities of the substance.  Table 8 shows the predictive targets of the bioactive compounds, isolated from *A. candolleanus*, identified through the STP software. Out of the four compounds tested, we found that scillirosidin and stemmadenine inhibited MAP kinase p38 alpha and beta pathways, responsible for an inflammatory imbalance during bacterial infections, whereas myxoxanthophyll and scillirosidin also target interleukin-8-receptor A and B, which are the major proinflammatory cytokines that get elevated in such patients. The PASS online predictivity scores for bioactive compounds isolated from the roots of *A. candolleanus* are reported in Table 9. The possibility that a chemical compound to be active (Pa) or inert (Pi) on a biological target is calculated using the PASS online software. The compounds having a Pa score of greater than 0.7 are considered highly active, while those having a Pa score greater than 0.3 are moderately active. Interestingly, the PASS analysis of the bioactive compounds (stemmadenine, myxoxanthophyll, cephalotaxine, and scillirosidin) revealed that myxoxanthophyll and cephalotaxine were predicted as apoptosis agonists, antioxidants, and anti-inflammatory with a Pa score above 0.7, for each biological activity (Table 9). Taken together, our results highlighted the prominent role of the isolate myxoxanthophyll which was found highly bioactive and therefore can be considered a promising candidate for drug design studies. From the overall results of drug design, we found that myxoxanthophyll is a promising bioactive isolate with high bioactivity.

## 4. Conclusions

The antibacterial activity of *A. candolleanus* extract was tested against several microbial strains, including *B. subtilis*, *S. aureus*, *E. coli*, *S. typhimurium*, *P. aeruginosa*, *K. pneumoniae*, and *K. rhizophila*, and results showed a broad inhibition zone against bacterial species. The activity may be a cumulative effect of all the constituents present in the plant. In our study, we have isolated four compounds that were identified as scillirosidin, cephalotaxine, stemmadenine, and myxoxanthophyll using FTIR and LC-MS-MS techniques. These compounds will be further tested individually against bacterial strains. We also performed computational studies to predict the most active constituent amongst all four compounds. The results of STP as well as PASS online predictivity score software testing showed that, among the four compounds, myxoxanthophyll was the most active molecule, revealing a predicted activity as an apoptosis agonist, antioxidant, and anti-inflammatory compound, with a Pa score above 0.7. However, this study can be considered a preliminary investigation of the chemical composition and biological activities of *A. candolleanus*. Further studies are required to validate the activity of these identified compounds using antimicrobial and docking studies.

## Figures and Tables

**Table 1:** Phytochemical screening for the presence of active constituents in roots of *A. candolleanus*.

Solvent	Alkaloids	Glycosides	Terpenoids	Flavonoids	Saponins	Carbohydrates	Proteins	Lipids	Volatile oils	Steroids	Phenols and tannins	Gums and mucilage
DMSO	−	−	−	−	−	−	−	+	+	−	−	−
*n*-Hexane	−	−	−	−	−	−	−	−	+	−	−	−
Petroleum ether	−	−	−	−	−	−	−	−	+	−	−	−
Chloroform	−	+	−	+	−	−	−	−	+	−	−	−
Methylene chloride	−	+	−	−	−	−	−	−	−	−	−	+
Acetone	−	−	−	−	−	−	−	−	+	−	−	+
Ethyl acetate	+	−	−	−	−	−	−	+	+	−	−	+
Methanol	+	−	−	−	−	−	−	+	+	−	−	+
Ethanol	+	−	−	−	−	−	−	+	+	−	−	+
Water	−	−	−	−	−	−	−	+	+	−	−	+
Methanol : water (7 : 3)	−	+	+	−	−	−	−	+	+	−	−	+
Butanol : acetic acid : water (4 : 1 : 5)	−	+	−	−	−	−	−	+	+	−	−	+

+: presence; −: absence.

**Table 2:** Antibacterial activity of *A. candolleanus* root extract and standard gentamycin (diameter inhibition zone expressed in mm).

Bacterial strains	DMSO	*A. candolleanus* extract (*µ*g/mL)					Gentamycin (*µ*g/mL)			
		5	10	20	40	80	5	10	20	40
*E. coli* ATCC 9637	0	21.2	22.7	23.5	24.2	25.4	11.6	12.6	13.2	14.8
*K. pneumoniae* ATCC 10031	0	19.7	21.8	—	—	—	12.6	12.9	16.7	21.5
*S. typhimurium* ATCC 1428	0	23.2	25.5	—	—	—	12.8	13.2	13.8	15.7
*P. aeruginosa* ATCC 25619	0	38.7	41.6	—	—	—	12.3	13.4	15.9	17.3
*S. aureus* ATCC 6538	0	26.3	28.6	29.7	30.4	32.7	12.4	14.4	15.2	15.8
*B. subtilis* ATCC 6633	0	37.5	41.2	—	—	—	18.9	19.8	21.9	22.4
*K. rhizophila* ATCC 9341	0	22.0	24.6	—	—	—	13.8	15.5	17.3	19.5

—: overlapping of zones.

**Table 3:** FTIR analysis of hydro-methanolic *A. candolleanus* root extract.

S. no.	Expected wave number (cm^−1^)	Observed wave number	Characteristic functional group	Compound type
1	2850–2970 1340–1470	1434.14 2878.88	C-H	Alkane
2	1050–1300	1067.65	C-O	Alcohol, ether, carboxylic acid, esters
3	1500–1570 1300–1370	1316.47 1354.09	NO_2_	Nitro
4	1610–1680	1628.95	C=C	Alkenes
5	1690–1760	1736.97 1751.44	C=O	Aldehyde, ketones, carboxylic acids, esters
6	3200–3600	3240.55	O-H	Phenols, hydrogen- bonded alcohols
7	3500–3650	3515.42	O-H	Monomeric carboxylic acids

**Table 4:** Mass spectral interpretation of peak 1 from LC-MS-MS done on *A. candolleanus* root extract.

S. no	Mass	Ion	Product ion and composition of neutral particle lost	Substructure or compound type	Specific *m/z* ratio
1.	1	−	[M − 1]^−^	Fragmented ion as base peak (hydride transfer peak occurs moderately basic and acidic compounds)	457.19

**Table 5:** Mass spectral interpretation of peak 3 from LC-MS-MS done on *A. candolleanus* root extract.

S. no	Mass	Ion	Product ion and composition of neutral particle lost	Substructure or compound type	Specific *m/z* ratio
1.	1	−	[M − 1]^−^	Fragmented ion as peak	314.15
2.	2	−	[M − 2]^−^	−	313.15

**Table 6:** Mass spectral interpretation of peak 4 from LC-MS-MS done on *A. candolleanus* root extract.

S.No	Mass	Ion	Product ion and composition of neutral particle lost	Substructure or compound type	Specific *m/z* ratio
1.	1	M^+^	[M − 1]^−^	Molecular ion fragmented ion as base peak	354.14
2.	23	Na^+^	[M − 23]^−^	Organic Na + salts	353.14
3.	41	C_3_H_5_^+^	[M − 41]^+^	Alicyclics (especially poly alicyclics) alkenes	331.16
		CH_3_CN^+^		2-Methyl-N-aromatics-N-methyl anilines	313.15

**Table 7:** Mass spectral interpretation of peak 5 from LC-MS-MS done on *A. candolleanus* root extract.

S.No	Mass	Ion	Product ion and composition of neutral particle lost	Substructure or compound type	Specific *m/z* ratio
1.	—	M^+^	—	Molecular ion	747.40
2.	1	—	[M + 1]^+^	Proton transfer ion	748.411

**Table 8:** Swiss Target Prediction for the bioactive isolate.

S. no.	Name of the compound	Target	Common name	UNIPROT ID	CHEMBL ID	Target class	Probability ^*∗*^	Known active (3D/2D)
1	Scillirosidin	MAP Kinase p38 alpha	MAPK14	Q16539	CHEMBL260	Kinase	0.100634432184	158/0
		MAP Kinase P38 beta	MAPK11	Q15759	CHEMBL 3961	Kinase	0.100634432184	176/0
		Interleukin-8 receptor B	CXCR2	P25025	CHEMBL 2434	Family AG-protein coupled receptor	0.100634432184	108/0
2	Cephalotaxine	Inhibitor of apoptosis protein 3	X1AP	P98170	CHEMBL 4198	Other cytosolic proteins	0.0	16/0
3	Stemmadenine	Inhibitor of apoptosis protein 3	X1AP	P98170	CHEMBL 4198	Other cytosolic proteins	0.109339753231	116/0
4	Myxoxanthophyll	Interleukin-2 (IL-2)	IL-2	P60568	CHEMBL5880	Secreted protein	0.428381527054	0/1
		Interleukin-8 receptor	CXCR1	P25024	CHEMBL4029	Family AG-protein coupled receptor	0.0	0/2

^*∗*^Probability for the query molecule assumed as bioactive to have this protein as target.

**Table 9:** PASS online predictivity score for bioactive compounds.

S. no	Name of isolated phytoconstituent	PASS (activity)/(inactivity) prediction score Pa^*∗*^ pi		Key mechanism of bioactivity
1	Scillirosidin (moderately active)	0.43200.33900. 25300.33700.1600	0.0590.0460. 0160.1310.017	Apoptosis agonist Antibacterial Transcription factor kappa B inhibitor Anti-inflammatory Cytokine release inhibitor
2	Cephalotaxine (high activity)	0.92300.19100.0660	0.0600.0040.058	Antioxidants Apoptosis agonist Glutathione reductase stimulant
3	Stemmadenine (moderately active)	0.37900.34200.35500.0970	0.830.621.190.87	Apoptosis agonistMAP3K5 inhibitorAnti-inflammatoryMAP kinase inhibitor
4	Myxoxanthophyll (high activity)	0.86600.82700.71700.21900.1230	0.050.0030.0140.0160.031	Apoptosis agonist Antioxidant Anti-inflammatory Interferon antagonist Cytokine release inhibitor

^*∗*^Pa > 0.7: highly active; Pa > 0.3: moderately active; Pa > 0.1: less active.

## Data Availability

The data presented in this study are available on request from the corresponding author.
